# Prevention of Protein Glycation by Natural Compounds

**DOI:** 10.3390/molecules20023309

**Published:** 2015-02-16

**Authors:** Izabela Sadowska-Bartosz, Grzegorz Bartosz

**Affiliations:** 1 Department of Biochemistry and Cell Biology, University of Rzeszow, Zelwerowicza St. 4, PL 35-601 Rzeszow, Poland; 2 Department of Molecular Biophysics, University of Lodz, Pomorska St. 141/143, 90-236 Lodz, Poland

**Keywords:** glycation, inhibitors of glycation, ageing

## Abstract

Non-enzymatic protein glycosylation (glycation) contributes to many diseases and aging of organisms. It can be expected that inhibition of glycation may prolong the lifespan. The search for inhibitors of glycation, mainly using* in vitro* models, has identified natural compounds able to prevent glycation, especially polyphenols and other natural antioxidants. Extrapolation of results of* in vitro* studies on the* in vivo* situation is not straightforward due to differences in the conditions and mechanism of glycation, and bioavailability problems. Nevertheless, available data allow to postulate that enrichment of diet in natural anti-glycating agents may attenuate glycation and, in consequence, ageing.

## 1. Glycation

Louis Camille Maillard (1912) first reported that reducing sugars react with amino acids in solution producing dark-colored products (melanoidins) [1]. Similar chemical reactions could be observed also in solutions of reducing sugars mixed with peptides and proteins. This reaction, called now the “Maillard reaction”, is a complex network of successive and parallel reactions. Initially,a sugar having the functionality of the aldehyde group, or an aldehyde, reacts non-enzymatically with a thiol or amino groups of a protein (or another biomolecule) forming a Schiff base. Comparison of reactivity of various amino acid residues in peptides with reducing sugars revealed the highest reactivity of side chains of cysteine, lysine, and histidine, and amino groups of the N-terminal amino acids [2]. The Schiff bases rearrange over a period of days to produce ketoamine or Amadori products. The Amadori products undergo dehydration and rearrangements followed by other reactions such as cyclisation, oxidation, and dehydration to form more stable Advanced Glycation End Products (AGEs) [3]. Dicarbonyl products, glyoxal and methylglyoxal, formed as intermediate products in the course of the Maillard reaction, are of great importance. These highly active compounds, which are also formed in the cell as by-products of glycolysis, can react with proteins producing cross-links resistant to the action of enzymes. Carboxymethyl-lysine (CML), a non-fluorescent protein adduct, was first described by Ahmed and represents the most prevalent AGE* in vivo* [4]. Pentosidine (a fluorescent glycoxidation product) was first isolated and characterized by Sell and Monnier [5]. Other AGEs identified* in vivo* include glucosepane, carboxymethyl-hydroxylysine, carboxyethyllysine (CEL), fructose-lysine, and pyrraline, which form non-fluorescent protein adducts, fluorescent methylglyoxal-derived hydroimidazolone (MGH1), which contributes significantly to skin fluorescence [6], and glyoxal-lysine dimer and methylglyoxal-lysine dimer forming non-fluorescent protein crosslinks [7]. Glycation alters the structure and functional properties of proteins, which affects adversely cellular metabolism.

AGE formation takes place under normal physiologic conditions but is accelerated in hyperglycemia [8,9,10]. AGEs may also form from non-glucose sources including lipid and amino acid oxidation [9,11,12]. Increased level of reactive oxygen species (ROS) cause oxidative stress; in analogy, increased concentration of sugars (glucose, deoxyglucose, fructose, ribose and triose phosphates) and active dicarbonyl compounds (glyoxal and methylglyoxal) can cause “carbonyl stress” resulting in the increased rate of formation of AGEs.

Among all the natural monosaccharides, glucose is characterized by the maximal shift of the equilibrium between the cyclic and aldehyde isoforms (only 0.2% existing in the aldehyde form). Thus, glucose is one of the least active sugars in relation to glycation and this property might have been the reason for the evolutionary choice of glucose as the universal carbohydrate energy carrier [13].

AGEs accumulate intracellularly mainly because of their generation from glucose-derived dicarbonyl precursors formed in the course of metabolism [14,15]. Although it is possible that intracellular AGEs can play positive roles as stimuli for activating intracellular signaling pathways and modifying the function of intracellular proteins, there is a plethora of evidence that their accumulation adversely affects protein structure and function. Cytoskeletal proteins are important in providing stability of the cytoskeleton and are crucially involved in numerous cellular functions such as migration and cellular division. Various other intracellular proteins including enzymes and growth factors may be targets of non-enzymatic modification by sugars. Glycated basic fibroblast growth factor (bFGF) displays impaired mitogenic activity in endothelial cells [14]. Glycation of enzymes of the ubiquitin-proteasome system and of the lysosomal proteolytic system has been shown to inhibit their action [16]. The structural components of the connective tissue matrix and basement membrane components (e.g., type IV collagen) as well as other long-lived proteins (including myelin, tubulin, plasminogen activator 1 and fibrinogen) can also undergo advanced glycation [17]. It should be mentioned that AGE-modified proteins may be more resistant to enzymatic degradation [18].

Accumulation of glycation products is associated with various diseases including, first of all, diabetes and diabetic nephropathy, microangiopathy and atherosclerosis [12]. Indeed, the intermolecular collagen cross-linking caused by AGEs leads to diminished arterial and myocardial compliance and increased vascular stiffness, phenomena that are considered to explain partly the increase in diastolic dysfunction and systolic hypertension seen in diabetic patients [19]. Yuen* et al.* (2010) suggested that collagen glycation augments the formation and migration of myofibroblasts and participates in the development of fibrosis in diabetes [20]. Other studies showed that glycated collagen alters the endothelial cell function and could be an important factor in atherosclerotic plaque development [21]. More recently, it was revealed that AGEs may act as mediators of the progression of stable to rupture-prone plaques; this finding opens a window towards biomarkers and novel treatments of cardiovascular diseases [22].

A significant effect of AGEs involves their interactions with receptors (receptors for AGEs or RAGEs and others). Interaction of AGEs with RAGEs generates secondary oxidative stress and plethora of other undesired effects including increased gene transcription of pro-inflammatory and pro-fibrotic cytokines and chemokines leading to an inflammatory condition [23], which, in turn, in many instances, promotes epithelial cell malignant transformation, contributing to tumorigenesis [24]. Shortened RAGEs (sRAGEs) circulating in blood and body fluids, which lack the transmembrane domain, include an endogenous secretory isoform generated via alternative splicing and a form generated through proteolytic cleavage of the full-length cell surface receptor, serve as competitive binding sites for AGEs diminishing their effects on RAGEs [12].

There is a relationship between activation of the AGE-RAGE system and some aspects of polycystic ovary syndrome (PCOS), such as granulosa cell dysfunction, adipocyte pathophysiology, obesity and insulin resistance. Furthermore, irregular ovarian AGE signaling might in part explain the abnormal ovarian histology observed in women with PCOS [25].

The serum level of AGEs has been found to be elevated in such diseases as cystic fibrosis [26], non-B or non-C hepatocellular carcinoma [27], relapsing-remitting multiple sclerosis [28,29] or schizophrenia [30]. It should be mentioned that glycation induces refolding of initially globular albumin into amyloid fibrils comprising cross-β structure [31]. Moreover, glycation induces the formation of the β-sheet structure in β-amyloid protein, β-synuclein, transthyretin, as well as copper–zinc superoxide dismutase. Aggregation of the β-sheet structure in the brain creates fibrillar structures, respectively causing Alzheimer’s disease, Parkinson’s disease, amyotrophic lateral sclerosis, familial amyloid polyneuropathy and prion disease. It has been also suggested that oligomeric species of glycated α-synuclein and prion are more toxic than fibrils [32].

A controversy remains regarding the content of AGEs as a biochemical marker of Alzheimer’s disease. Thome and co-workers (1996) examined the question whether the reported increased level of AGEs in the brain is reflected in an increase in AGE-associated parameters in peripheral blood [33]. These authors reported that elevated central nervous system AGEs levels in patients with Alzheimer’s disease are manifested without detectable peripheral changes, however other investigators demonstrated moderate increases in Amadori products of plasma proteins [34]. A more recent study revealed a lower level of circulating serum AGEs in patients with Alzheimer’s disease in relation to healthy controls [35].

In addition, literature data indicate that accumulation of AGEs plays an important role in the development of degenerative changes in the lens of the eye, leading to blindness or cataract. Progression of cataract is increased in patients with diabetes mellitus [36]. AGEs induce irreversible structural changes in the protein, resulting in the formation of protein aggregates of high molecular weight, which impede vision and light scatter [37]. It has been shown that AGEs, by altering the surface charge of the protein, lead to conformational changes and consequently reduces the transparency of the lens of the eye [38]. AGEs also play an important role in the progression of diabetic retinopathy leading to dysfunction or death of retinal cells [39]. Recent reports indicate that detoxification of methylglyoxal reduces the accumulation of AGEs, which in turn can prevent the pathological changes in the retina and vessels [40].

## 2. Glycation and Ageing

Glycation has been repetitively proposed to contribute to the process of ageing. Accumulation of products of protein glycation with ageing has been observed [8,41]. In the early 1980s, after AGEs had been found to accumulate with age in tissues of living organisms, a theory of “non-enzymatic glycosylation as the cause of ageing” was proposed [42]. Many age-related deteriorative changes are actually due to protein degradation, such as posttranslational modification, accumulation of molecular waste, deterioration of functional proteins, functional disorders of the tricarboxylic acid cycle, or activation of inflammatory pathways by intracellular signals. All of these changes are symptomatic of “glycation stress” [43]. AGEs are metabolized by protease and oxidized protein degradation enzymes, such as oxidized protein hydrolase, in the proteasome and then excreted [44]. Other enzymes are also known to modify AGEs and intermediate compounds. For example, glyoxalase 1 is the key enzyme that converts the highly reactive α-oxo-aldehydes into the corresponding α-hydroxy acids using l-glutathione as a cofactor. Unfortunately, the activity of proteolytic enzymes decreases with age [41,45]. Most recent studies revealed that AGEs are mitogenic compounds and trigger cell cycle reentry of neurons in Alzheimer’s disease brain. The reduction of oxidative stress by application of α-lipoic acid decreased AGEs accumulations, and this decrease was accompanied by a reduction in cell cycle reentry and a more euploid neuronal genome [46].

Galactose is much more effective than glucose as a glycating agent amount of aldehyde for many times exceeds that of glucose [47]. Addition of galactose to the diet was shown to cause typical premature ageing, in which the mitochondrial path of apoptosis involving cytochrome *c* release from mitochondria plays an important role [48]. This effect was attenuated by salidroside, an inhibitor of RAGE-type receptors [49]. Metformin, inhibiting AGEs formation from monosaccharides, is also known to be a geroprotector [2].

At the cellular level, aminoguanidine was shown to increase the replicative lifespan of human lung fibroblasts from 54 up to 75 population doublings (at 4 mM aminoguanidine) and decreased the rate of telomere shortening by more than 50%. While several mechanisms can contribute to this effect, the inhibition of glycation may be significant [50]. It has been postulated that accumulation of AGEs is the basis of “biological clock” governing ontogenesis and ageing [2].

Ageing is associated with a chronic low-grade inflammatory status that contributes to chronic diseases such as age-related muscle wasting, kidney disease, and diabetes mellitus. AGEs are known to be proinflammatory. Intervention studies in humans showed mainly a decrease in inflammation in subjects on a low-AGE diet, while an increase in inflammation in subjects on a high-AGE diet was less apparent [51]. About half of the observational studies found a relationship between inflammatory processes and AGEs in food. The dietary intake of AGEs appears to be related to inflammatory status and the level of circulating AGEs. Limiting AGE intake may lead to a decrease in inflammation and chronic diseases related to inflammatory status [51]. Moreover, lowering the content of AGEs in the normal diet significantly prevents AGEs accumulation, attenuates oxidative stress and extends lifespan in mice. In humans, short-term trials demonstrated that a low-AGEs diet reduces oxidant burden and inflammatory markers [8].

## 3. Inhibition of Glycation

Preventive medicine seems to be the best approach to preventing the development of lifestyle-related diseases such as atherosclerosis and diabetic complications. The daily intake of AGEs inhibitors in natural products can play a beneficial role in preventing the pathogenesis of lifestyle-related diseases. Therefore, natural compounds have been screened as potential inhibitors of AGEs formation [52].

A class of compounds is known that prevent the formation of AGEs or degrade the existing AGEs. Some of them have been produced and patented. They include: First of all, aminoguanidine and anti-type 2 diabetes drugs such as metformin and pioglitazone (patented). From among other drugs in use, angiotensin receptor blockers, inhibitors of angiotensin converting enzyme, and pentoxyfylline (patented) were also found to inhibit AGE formation. Other inhibitors of protein glycation include antioxidants, such as vitamin C and vitamin E, and metal ion chelators (desferoxamine and penicillamine). Aspirin inhibits glycation competitively by capping amino groups. Amadori products already formed may be deglycated by enzymes called amadoriases. A group of compounds has been discovered, which break α-dicarbonyl cross-links, among them phenacylthiazolium bromide and its stable derivative ALT-711 (Alagebrium). Finally, derivatives of aryl ureido and aryl carboxaminido phenoxy isobutyric acids (patented) protect against glycation. Some of these compounds such as metformin, pioglitazone, pentoxyfylline and aspirin have already been used in clinical practice, some (aminoguanidine and ALT-711) have been tested in clinical trials [53].

Aminoguanidine was the first AGEs inhibitor discovered in 1986; its mechanism of action involves catching reactive intermediates generated by the Maillard reaction. Animal models of type 1 and 2 diabetes showed that aminoguanidine prevents formation of AGEs and thus diabetic complications, including vascular ones [54]. Aminoguanidine half-life in plasma is short (about 1 h), therefore, it must be applied at a relatively high dose (1 g/L of water) to produce a concentration sufficient to trap reactive species [55]. The use of high concentrations of aminoguanidine is not preferred because of its reaction with vitamin B6, which in turn causes a deficiency. Another inhibitor of AGE formation is pyridoxine; its mechanism of action involves blocking the oxidation of compounds formed by the Maillard reaction, catching of reactive oxygen species and reactive carbonyl and dicarbonyl compounds or metal chelates which catalyze the oxidation reactions [56]. Just as aminoguanidine, pyridoxine exhibits inhibitory effect of vascular diseases associated with diabetes, in addition to lowering cholesterol and triglyceride levels [57]. Among other substances acting on the basis of catching reactive carbonyl compounds 2,3-diaminophenazine and penicillamine should be mentioned. However, to date, there are no studies* in vivo* identifying the reaction products of these substances with the compounds produced during the process of glycation. Thiamine and benfotiamine one have also been described as inhibitors of the formation of AGEs, but their action is limited [58]. Based on the structure of thiamine and benfotiamine one can speculate that they may be effective metal chelators. It seems that research should focus on the selection of those substances that are both inhibitors of AGEs and will chelate metal ions effectively, are nontoxic, and their half-life* in vivo* is quite long.

There is a big interest in the search of compounds of natural origin which can inhibit glycation, apart from those already mentioned. Search for such compounds is based, in the first stage, on* in vitro* screening experiments. However, results of* in vitro* experiments may be misleading due to several reasons. Most AGEs products are formed by glycooxidative mechanisms that require oxygen and are catalyzed by traces of redox active transition metal ions [59,60]. *In vitro* assays for AGE formation and inhibition cannot adequately mimic the metal ion distribution or antioxidant and detoxification mechanisms in tissues and their various compartments. Especially, the sugar concentration and oxygen pressure are usually much higher in the* in vitro* experiments than* in vivo*. Autooxidation of glucose (Wolff pathway) or Schiff bases (Namiki pathway) may dominate at high glucose* in vitro* but not at low glucose and high oxygen level* in vivo* [60]*.*

The complex nature of the Maillard reaction makes it difficult to identify the mechanism of inhibition of glycation. Khalifah* et al.* proposed a procedure to prepare proteins rich in Amadori compounds but free from AGEs (“pre-glycated” albumin) to identify compounds inhibiting glycation at post-Amadori stage of AGE formation (amadorins). Pyridoxamine, in contrast to aminoguanidine, was found to have amadorin activity [60]*.*

## 4. Inhibition of Glycation *in Vitro*

The antiglycation properties of numerous medical herbs and dietary plants are of a similar [61] or even higher order [62,63] than that of standard inhibitor of glycoxidation - aminoguanidine. In an* in vitro* assay, methanol extracts of whole plants of *Calendula officinalis* and fruits of *Juglans regia* showed antiglycating activity with respect to bovine serum albumin (BSA) comparable to that of aminoguanidine on the weight concentration basis [61]. 16 compounds were isolated from ethyl acetate extracts of *Erigeron annuus*, 3,5-di-*O*-caffeoyl-*epi*-quinic acid being the most active inhibiting BSA glycation, preventing opacification of lenses and inhibiting aldose reductase [63]. Ethanol extracts of 14 wild berries were compared for their antiglycating activity* in vitro*. Extract of *Empetrum nigrum* L. showed the strongest activity; the anti-glycating activity correlated with the radical scavenging activity of the extracts [64]. Comparison of antiglycating activity of eight anthraquinones from the roots of *Knoxia valerianoides* showed considerable activity of lucidin and 1,3,6-trihydroxy-2-methoxymethylanthraquinone [65]. Maltol was also found to have a stronger* in vitro* AGE inhibiting activity compared with aminoguanidine [62].

*In vitro* glycation assays showed that a number of polyphenols exerted inhibitory effects on the glycation reaction. Polyphenols are the most abundant antioxidants in our diets. The main classes of polyphenols are phenolic acids (mainly caffeic acid) and flavonoids (the most abundant in the diet are flavanols, especially catechins plus proanthocyanidins), anthocyanins and their oxidation products), which account for one- and two-thirds of dietary polyphenols, respectively. Polyphenols are reducing agents, and together with other dietary antioxidants, such as vitamin C, vitamin E and carotenoids, protect the body’s tissues against oxidative stress and associated pathologies such as cancers, coronary heart disease as well as inflammation [66]. Comparison of the anti-glycating activity* in vitro* of ethanol/water extracts of coriander, turmeric, scallion, pepper mint, onion, parsley, ginger, curry, scallion, pepper mint, onion and parsley leaves showed a good correlation between the anti-glycating and antioxidant activities of the extracts [67].

Phenolic acids are the main polyphenols made by plants. These compounds have diverse functions and are immensely important in plant-microbe interactions/symbiosis. Adisakwattana* et al.* (2012) found that cinnamic acid and its derivatives could effectively protect BSA from fructose-mediated protein glycation* in vitro* [68]. Recent investigations suggested that cinnamic acid derivatives such as ferulic acid (3-methoxy-4-hydroxycinnamic acid) and isoferulic acid (3-hydroxy-4-methoxycinnamic acid), which are the main active components of the rhizoma of *Cimicifuga heracleifolia*, an anti-inflammatory drug used frequently in Japanese traditional medicine, are also AGEs inhibitors [69,70,71]. The results obtained by Srey* et al.* (2010) indicated that ferulic acid effectively inhibits CML and CEL formation in model food systems [72]. Silván* et al.* (2011) reported that ferulic acid at a final concentration of 2.5 mg/mL exerts a clear anti-glycation effect, mainly due to an inhibition of the advanced stage of the glycation reaction (specific anti-AGE effect) [69]. More recently Meeprom* et al.* (2013) showed that isoferulic acid (1.25–5 mM) inhibits the formation of fluorescent AGEs and non-fluorescent AGE (CML) and fructosamine protein adducts [71]. It should be noted that isoferulic acid has been found to be a metal ion chelating agent. From this point, metal chelating activity of isoferulic acid might be one of possible mechanism responsible for inhibition of glycation.

Huang* et al.* (2008) investigated the inhibitory abilities of phenolic acids (chlorogenic acid, syringic acid and vanillic acid) on methylglyoxal-induced mouse Neuro-2A neuroblastoma (Neuro-2A) cell apoptosis in the progression of diabetic neuropathy. The data indicated that methylglyoxal induced mouse Neuro-2A neuroblastoma (Neuro-2A) cell apoptosis via alternation of mitochondria membrane potential and Bax/Bcl-2 ratio, activation of caspase-3, and cleavage of poly (ADP-ribose) polymerase. Moreover, the results showed that activation of mitogen-activated protein kinase signal pathways (JNK and p38) participated in the methylglyoxal-induced Neuro-2A cell apoptosis process. Thus, treatment of Neuro-2A cells with phenolic acids suppresses cell apoptosis induced by methylglyoxal, suggesting that phenolic acids possess cytoprotective ability in the prevention of diabetic neuropathy complications [73].

Other polyphenols present in many dietary sources also have the anti-glycating activity. For example, ellagic acid (2,3,7,8-tetrahydroxy-chromeno[5,4,3-cde]chromene-5,10-dione) is one of the commonly found dietary polyphenols. Apart from the greatest sources, such as berries and pomegranate, ellagic acid is also present in apples, grapes, orange, guava and cumin. Ellagic acid is known to have antioxidant, anti-inflammatory and anticarcinogenic properties. The antiglycating action of ellagic acid seems to involve, apart from inhibition of a few fluorescent AGEs, predominantly inhibition of CEL through scavenging of the dicarbonyl compounds. Furthermore, MALDI–TOF-MS (matrix assisted laser-desorption ionisation–time-of-flight MS) analysis confirms inhibition of the formation of CEL on lysozyme on* in vitro* glycation by ellagic acid. Prevention of glycation-mediated β-sheet formation in hemoglobin and lysozyme by ellagic acid confirm its antiglycating ability [74].

Gugliucci* et al.* (2009) evaluated the anti-glycation effect of some bioactive substances present in yerba maté (*Ilex paraguariensis*): 5-caffeoylquinic acid, caffeic acid and sapogenin (oleanolic acid). These authors suggested that chlorogenic acid and caffeic acid are the main substances responsible for the anti-glycation effect of maté tea [75]. Chlorogenic acid is a phenolic compound formed by the esterification of caffeic and quinic acids. The inhibitory effects of chlorogenic acid on AGEs formation and collagen cross-linking may be caused by its interactions with reactive dicarbonyl compounds, such as methylglyoxal. Chlorogenic acid could be expected to be beneficial in the prevention of AGEs progression in patients with diabetes [76]; however, no results clinical studies with chlorogenic acid have been published.

Flavonoids (a diverse class of polyphenolic compounds), have been also demonstrated to be effective inhibitors of glycoxidation. The inhibition of glycoxidation has been showed for various polyphenols, including quercetin, genistein, tannic acid and gallic acid [77,78,79,80,81]. Our recent study* in vitro* on prevention of BSA glycation showed that the same compounds were found to have different effects on glycoxidation induced by various sugars, glyoxal and methylglyoxal, which suggests caution in extrapolation from experiments based on one sugar to other sugars. From among the compounds tested, the most effective inhibitors of glycoxidation were: polyphenols, pyridoxine and 1-cyano-4-hydroxycinnamic acid. As standard antioxidants had a stronger effect than metal chelators, we ascribe the inhibition of BSA glycation by polyphenols mainly to their antioxidant rather than metal-chelating properties [82,83].

Xie and Chen (2013) summarized the structural features of flavonoids relevant for their anti-glycation activity. They concluded that: (i) The hydroxylation on both A ring and B ring improved the inhibitory activity on AGEs formation, while hydroxylation on C ring decreased the activity; (ii) The methylation generally reduced the anti-AGEs activity of flavonoids, except for the 3-O-methylation of flavonols; (iii) The glycosylation of hydroxyls of flavonoids tended to decrease the inhibitory activities on inhibiting AGEs formation, although contradictory results were also reported; (iv) Hydrogenation of the C2=C3 double bond of flavones slightly weakened their activities; (v) A 5,7-dihydroxy structure was favorable; (vi) Proanthocyanidins dimers or trimers showed a stronger inhibitory activity than catechins, and the glucosides of anthocyanidin had higher activities than their rutinosides; (vii) The hydroxylation on B ring and the methylation of stilbenes decreased the inhibitory activity; (viii) The presence of galloyl groups was important for the activity of catechins, and α-hydroxyl group at C-3 was much more effective than β-hydroxyl group at C-3; (ix) The phenolic acids with multiple hydroxyls showed strong inhibition of AGEs formation, and an *ortho* or *meta* dihydroxyl structure on the benzene ring was vital to the anti-AGEs activity of anthraquinones; (x) Both ellagic acids and ellagitannins showed potent inhibitory activities on AGEs formation, and hydroxylation increased the activities but methylation decreased them [80]. Components of animal-derived diet may also have anti-glycating properties. Carnitine was found to be an effective anti-glycating compound both* in vitro* and* in vivo* [84].

The above results suggest that consumption of a polyphenol-rich diet may attenuate protein glycation to some extent, and the addition of polyphenols can be useful in reducing undesired glycoxidation in food processing.

## 5. Inhibition of Glycation *in Vivo*

### 5.1. Reduction of AGE Intake

Dietary AGEs constitute a significant source of AGEs in the body. AGEs formation can be rapidly accelerated by increasing the time and degree of exposure to heat and can be introduced into the body in heat-processed foods. E. g., pretzel sticks are a rich source of pentosidine and pyrraline [85]. AGEs are also present in the cigarette smoke, are inhaled into the alveoli, and then they are transported to blood stream or to lung cells where they can interact with other glycation products and contribute to protein glycation [86]. While detailed mechanisms of intestinal absorption of AGEs are not fully elucidated, it is known, e.g., that pyrraline is absorbed by the peptide transporter hPEPT1 [87]. It has been estimated that ca 10% of ingested immunoreactive AGEs are transported into circulation, two-thirds of which remain in the body. Exogenous AGEs are incorporated covalently in tissues, and only one third is excreted via the kidneys [88]. A significant correlation between the amount of ingested AGEs and the plasma levels of these compounds was found in humans [89].

It has been controversial whether dietary AGEs are harmful to human health, because these compounds are heterogeneous and only a few have been characterized. Some of the products formed during this intricate reaction are furfurals, pyrralines and dicarbonyl compounds such as methylglyoxal. The products formed in the last reaction of this process are known as melanoidins in food science. CML has been reported as one of the most abundant* in vivo* and it was one of the first to be characterized in foods (milk and milk products). For this reason in most studies CML is chosen as a marker of AGEs in foods and* in vivo* [90]. However, long-term consumption of AGEs in rats was found to increase the levels of fasting glucose, insulin and serum AGEs [91], and induced a dose-dependent increase in proteinuria that over time could induce renal damage [92]. In mice, reduced dietary AGEs have been found to attenuate insulin resistance, increase the prevention of diabetes and, in diabetic mice, reduce diabetic vascular and renal complications, and improve impaired wound healing [93].

It should be mentioned that human breast milk contains approximately 70-fold lower amounts of CML than commercial infant milk and breast-fed infants had significantly lower plasma CML compared to infants fed with commercial infant milk [94].

Human studies demonstrated that intake of dietary AGEs by people with type 1 and 2 diabetes promotes the formation of pro-inflammatory mediators, leading to tissue injury [95]. Patients with uremia, with and without diabetes, in whom the intake of AGEs was reduced, showed reduced levels of inflammatory molecules such as TNF-α and high sensitivity C-reactive protein (hsCRP) [96]. In another study in patients with type 2 diabetes mellitus, decreasing the intake of AGEs for six weeks resulted in decreased levels of circulating AGEs and inflammatory markers [97]. The effects of reducing dietary AGEs have also been studied in nondiabetic peritoneal dialysis patients, a group that has very high AGE levels, and the results showed significant reduction in the levels of AGEs and C-reactive protein [96].

The positive effects of calorie restriction on the lifespan of various animals, especially rodents, are well known, though the generality of this phenomenon has been recently questioned [98]. Calorie restriction involves decrease in the AGE intake. It is possible that reduction in AGE consumption contributes significantly to the beneficial effects of calorie restriction [99].

All these data suggest that reduction of dietary intake of AGEs and reduction or elimination of smoking can contribute to lowering the level of AGEs in the body.

### 5.2. Effect of Exogenous Compounds of Natural Origin

#### 5.2.1. Problems with Exogenous Modification of Glycation

Prevention of glycation* in vivo* is not easy to achieve. Most drugs are enzyme inhibitors of receptor ligands and usually have half-maximal activity at nanomolar to micromolar concentrations. In contrast, glycation inhibitors must react stoichiometrically with low molecular mass, soluble, reactive intermediates of the AGE formation pathway in the presence of much higher concentrations of reactive functional groups on proteins. Lysine, for example, which is a major site of chemical modification of proteins by AGEs, is present in plasma proteins at a concentration of nearly 50 mM. An AGE inhibitor, which is unlikely to achieve a concentration of even of 100 μM in plasma, should be significantly more reactive with intermediates of AGE formation than protein lysine and other reactive residues [100]. Alternatively, the inhibitor may intercept the formation of AGEs at a stage either preceding the formation of the reactive carbonyl intermediates or after formation of a reactive adduct with protein. At the same time, it must display these activities without interfering with the intermediary metabolism of aldehydes or ketones, or trapping coenzymes or their precursors that contain reactive aldehydes, such as pyridoxal phosphate and retinal [101]. Bioavailability of exogenous compounds is an important problem in dietary interventions. Vlassopoulos* et al.* (2014) carried out a systematic literature review of dietary interventions reporting plasma concentrations of polyphenol metabolites. High dietary polyphenol intake, 3-hydroxyphenylacetic acid is the most abundant phenolic acid in peripheral blood (up to 338 μM) with concentrations of other phenolic acids ranging from 13 nM to 200 μM [102].

#### 5.2.2. Effects of Natural Compounds

Pyridoxamine (1 g/L drinking water) retarded the development of renal disease, measured by increases in urinary albumin and total protein and plasma creatinine, in the streptozotocin (STZ)-induced diabetic rat and inhibited a ca 2-fold increase in CML, CEL, Maillard-type fluorescence, and crosslinking of skin collagen of diabetic rats, compared to non-diabetic controls, after 7 months of diabetes [57]. Another study employing 0.4 g/L in drinking water, inhibited modest increases (20%–25%) in MOLD (methylglyoxal–lysine dimer) and pentosidine concentration in plasma proteins, and also inhibited a nearly 3-fold increase in plasma and erythrocyte methylglyoxal concentrations [102]. Pyridoxamine retarded also the development of retinopathy in the STZ-diabetic rat, as measured by protection against pericyte loss and formation of acellular capillaries [103]. In Zucker obese (fa/fa) rats, pyridoxamine inhibited the increases in fluorescence, CML, and CEL in skin collagen, increase in malondialdehyde and hydroxynonenal, and retarded early retinopathy as judged from proteinuria and plasma creatinine [104]. A study of the effect of 15 natural flavonoids, stilbenes and caffeic acid oligomers pointed to significant inhibition by all the flavonoids tested, especially hesperidin, naringin, quercetin and kaempferol. Resveratrol, piceatannol, epirabdosin, lithospermic acid and lithospermic acid B had also anti-glycating activity similar to aminoguanidine [105]. However, polyphenols act also via interference with RAGE signaling; this effect may contribute to the antitumor activity of polyphenols [106] so the effects observed may be contributed by other mechanisms irrespective of inhibition of glycation.

Anti-glycating effect may be simply due to lowering of blood glucose level; such action has been demonstrated, e.g., for methanolic bark extract of *Albizia odoratissima* Benth. [107].

Recently Li* et al.* (2014) employed a d-galactose-induced ageing rat model to investigate the protective effect of the saponins from *Aralia taibaiensis* (*Araliaceae*). They suggested that, by activating AKT/Forkhead box O3a and nuclear factor-erythroid 2-related factor 2 pathways, saponins supplementation increased the expression and function of their downstream antioxidants, including superoxide dismutase 2, catalase, glutathione reductase, glutathione, glutamate-cysteine ligase, and heme oxygenase 1, at least in part contributing to the protection against d-galactose-induced ageing [108]. It has been also found that the oral administration of Asn-Trp or carnosine (β-alanyl-l-histidine) dipeptides ameliorates oxidative stress and learning dysfunctions in d-galactose-induced ageing BALB/c mice [109].

Prisila Dulcy* et al.* (2012) examined the neuroprotective effect of standardized *Bacopa* *monniera* extract (BME: BESEB CDRI-08) against the d-galactose (d-gal)-induced brain ageing in rats. These findings suggest that BME treatment attenuates d-gal-induced brain ageing and regulates the level of antioxidant enzymes, NF-E2-related factor 2 expression, and the level of serotonin, which was accompanied by concomitantly increased levels of the presynaptic proteins (synaptotagmin I, synaptophysin) and the postsynaptic proteins (Ca^2+^/calmodulin dependent protein kinase II) as well as postsynaptic density protein-95 [110].

The administration of 50 mg/kg per day of maltol suppressed the elevated serum levels of glycosylated protein, renal fluorescent AGEs, CML, receptors for AGEs and nuclear factor-kappaB p65 in diabetic control rats, and protected against renal damage [62]. However, in another study 500 mg/kg/day epicatechin enhanced rather than decreased the CML accumulation on the surface of gastric epithelial cells in streptozotocin-diabetic mice [111].

Examples of more detailed results of* in vivo* studies are shown in Table 1.

Stem cells have various potential uses in most medical areas due to their differentiation and paracrine effects. Zhang* et al.* (2014) reported that adipose-derived stem cells (ASCs) provide a functional benefit by glycation suppression, antioxidation, and trophic effects in a mouse model of ageing induced by d-galactose. They showed that ASCs can decrease the AGE level, therefore reversing the ageing phenotype, which is a similar effect to that of aminoguanidine, and inhibitors of AGEs and ASCs can decrease the expression of senescence-associated markers such as superoxide dismutase and malondialdehyde [112].

#### 5.2.3. Effects of AGE Breakers

AGE breakers, a new class of candidate drugs targeting ageing-related cardiovascular dysfunction, may be useful as novel adjuvant agents to improve the efficacy of diabetic hypertension treatment. Experiments conducted by Zhang et al. (2014) demonstrated that 4,5-dimethyl-3-phenacylthiozolium chloride (alagebrium, ALT-711) significantly improves the anti-hypertensive actions of nifedipine, a Ca^2+^ channel blocker, in a rat model of streptozotocin-induced diabetic hypertension [113]. Freidja et al. (2014) reported that ALT-711 did not improve flow-mediated remodeling of resistance arteries in mature Zucker Diabetic Fatty rats but it reduced oxidative stress and consequently improved endothelium-dependent relaxation. On the other hand, in mature lean Zucker rats, ALT-711 improved flow-mediated remodeling of resistance arteries and reduced AGEs level. Thus, AGEs breaking, at least using ALT-711, could be a useful therapeutic tool in ameliorating diabetic complications and with the capacity to improve flow-mediated remodeling in non-diabetic subjects [114]. AGEs breaking and antioxidant treatment improves endothelium-dependent dilation without effect on flow-mediated remodeling of resistance arteries in old Zucker diabetic rats. Sakul* et al.* (2013) applied 2-ethoxycarbonyl-8-methoxy-2,3,4,4a,5,9b-hexahydro-1H-pyrido[4,3-b]indolinium dichloride (SMe1EC2) treatment during 4 months to aged streptozotocin-diabetic rats. They demonstrated that AGEs and 4-hydroxy-nonenal-histidine levels is significantly elevated in brain, ventricle and kidney, but not in lens and liver of aged rats when compared with young rats. In aged diabetic rats, SMe1EC2 protected only the kidney against increase in AGEs. However, it is not certain whether any natural compounds can act as AGE breakers [115].

**Table 1:** Results of chosen* in vivo* studies of the effects of natural compounds on glycation.

Population	Intervention	Main Findings	Reference
Healthy male Sprague–Dawley rats (220 ± 20 g) were divided randomly into four groups each containing 10 rats: control group, fructose group, betanin 25 mg/kg per day group, and betanin 100 mg/kg per day group.	Fructose water solution (30%) was accessed freely, and betanin (betanidin 5-*O*-β-d-glucoside, red) (25 and 100 mg/kg/d) was administered by intra-gastric gavage continuously for 60 days.	Betanin decreased protein glycation indexed by the relative lower methylglyoxal/ N-carboxymethyl lysine (CML) level and RAGE expression, and reduced glycative products in BSA/fructose system. Betanin also antagonized oxidative stress and NF-κB activation, all of them may be involved in the antifibrotic mechanisms. Food pigments may neutralize adverse effects of carbohydrate,* i.e.*, diabetes and related syndrome, and complementary therapy with betanin may prove useful in attenuating the development of cardiac fibrosis in diabetes.	[116]
Male C57 BLKS/J genetic background (*db/db*) mice and their non-diabetic lean littermates (*db/m*; 6-wk-old)were randomly divided into five groups (*n* = 8 each).	Mice were orally administered vehicle (sterile distilled water), metformin (300 mg/kg), and (+)-catechin (15, 30, and 60 mg/kg fresh preparation with sterile distilled water) daily at 4:00 pm, continuously for 16 weeks. Metformin was used as a positive antidiabetic drug, and *db/m* mice were used as non-diabetic controls. After 4–6 h fasting at the end of the treatment period, mice were killed and kidney tissues were saved for further assays.	(+)-Catechin might ameliorate renal dysfunction in diabetic mice as consequences of inhibiting AGEs formation and cutting off inflammatory pathway via methylglyoxal trapping.	[117]
Two-month-old male Wistar NIN rats with an average bodyweight of 220 ± 17 g were used in the study. Animals were distributed into four groups (groups I–IV). Each group consists of six animals.	All the animals were fed with AIN-93 diet *ad libitum*. The control (group I) rats received sham consists of 0.1 M citrate buffer, pH 4.5 while the experimental rats received a single i.p injection of streptozotocin (STZ, 35 mg/kg) in citrate buffer. Animals in group II received AIN-93 diet alone whereas group III animals received the AIN-93 diet supplemented with 3% cinnamon powder whereas group IV animals received AIN-93 diet containing 0.002% procyanidin-B2 -fraction. All the animals had free access to water.	Supplementation of diabetic rats with cinnamon and procyanidin-B2 -fraction prevented glycation mediated RBC-IgG cross-links and HbA1c accumulation in diabetes rats. Cinnamon and procyanidin-B2 -fraction also inhibited the accumulation of CML, a prominent AGE in diabetic kidney. Cinnamon and its procyanidin-B2 -fraction prevented the AGE mediated loss of expression of glomerular podocyte proteins; nephrin and podocin. Inhibition of AGE by cinnamon and procyanidin-B2 -fraction ameliorated the diabetes mediated renal malfunction in rats as evidenced by reduced urinary albumin and creatinine. Procyanidin-B2 from cinnamon inhibited AGE accumulation in diabetic rat kidney and ameliorated AGE mediated pathogenesis of diabetic nephropathy.	[118]
Male Wistar rats (200–230 g) were obtained from Sanzyme Ltd. (Hyderabad, India). The animals were divided into 4 groups (*n* = 8).	Diabetes was induced in all the male Wistar rats (200–250 g) except a group of eight animals which were treated as naïve (group I) by intraperitoneal administration of STZ (45 mg/kg) dissolved in freshly prepared citrate buffer (pH 4.5). The animals were fasted for 12 h before STZ administration and supplemented with 10% glucose for 48 h after STZ administration. One week after streptozotocin administration, blood glucose was estimated and the animals with more than 300 mg/dL were treated as diabetic and after a period of 6 weeks, the animals were divided into 3 groups. Group II served as diabetic control where as group III and group IV received resveratrol (10 mg/kg) and fidarestat (1 mg/kg), by per oral administration respectively, for a period of 3 weeks.	Resveratrol significantly improved glycaemic status and renal function in diabetic rats with a significant decrease in the formation of AGEs in the kidneys.	[119]
Male Wistar rats (initial weight of 50–75 g) were obtained from Charles River Breeding Laboratories (St-Constant, Qc, Canada). The animals were divided into six groups (Ctr_3,_ *n* = 10; D_3_, *n* = 8_; _PYR, *n* = 8; Ctrl_7_, *n* = 9; D_7_, *n* = 8; ALA, *n* = 7).	Rats were fed a high fat diet containing rodent diet D12451 (45 kcal % fat, 35 kcal % carbohydrates and 20 kcal % protein; Research Diets, New Brunswick, NJ, USA) *ad libitum* during 8 weeks, followed by a single dose of STZ (30 mg/kg intra-peritoneally). Four weeks after the injection of STZ, rats received warfarin (20 mg kg^−1^ day^−1^ in drinking water) and vitamin K (phylloquinone, 15 mg kg^−1^ day^−1^ sub-cutaneous injection, Spectrum Chemical, New Brunswick, NJ, USA) during 3 (D_3_), 5 (D_5_) and 7 (D_7_) weeks. To determine the implication of AGEs in initiating elasto-calcinosis, a subgroup of D_3_ rats received pyridoxamine (200 mg. kg^−1^ day^−1^) in powdered chow starting the same day as the STZ injection (thus during 7 weeks, including the 3 weeks of warfarin vitamin K (WVK) treatment) to prevent AGEs formation (group labeled PYR). To study the role of AGEs later in the calcification process, alagebrium (10 mg. kg^−1^ day^−1^, Synvista, Montvale, NJ, USA) was introduced in the food 7 weeks after the STZ injection (after 3 weeks of WVK treatment) and rats studied 4 weeks later (group labeled ALA). Dosages were adjusted every second day according to food intake. Controls consisted of age-matched untreated rats (Ctrl_3_ or Ctrl_7_).	Pyridoxamine (PYR) prevented AGE accumulation, whereas alagebrium chloride (ALT-711) induced a regression of AGE cross-links. PYR prevented calcium accumulation, while alagebrium blunted the progression of calcification.	[120]
Sprague Dawley (SD) rats were divided into five groups (*n* = 9 each).	Diabetes was induced by a single injection of streptozotocin (STZ, 60 mg/kg, intraperitoneally) in rats. Age-matched control rats (aged 6 weeks) received an equal volume of vehicle (0.01 M citrate buffer, pH 4.5). To investigate the effects of *Cassiae semen* (CS) extract, treatment was begun one week after the onset of diabetes and the compound was orally administered to the rats once a day for 12 weeks. SD rats were divided into groups: (1) normal rats (N), (2) normal rats treated with CS (N + CS), (3) STZ-induced diabetic rats (DM), (4) STZ-induced diabetic rats treated with CS (DM + CS, 100 mg/kg body weight), and (5) STZ-induced diabetic rats treated with aminoguanidine (AG), a positive control for AGEs inhibitor (DM + AG, 100 mg/kg body weight).	Oral treatment of CS can inhibit the development of diabetic nephropathy via inhibition of AGEs accumulation in STZ-induced diabetic rats The CS-treated group had significantly inhibited COX-2 mRNA and protein, which mediates the symptoms of inflammation in the renal cortex of diabetic rats. Histopathological studies of kidney tissue showed that in diabetic rats, AGEs, the receptor for AGEs, TGF-β1, and collagen IV were suppressed by CS treatment.	[121]
*In vivo* experiments were performed on 6-week-old male Wistar albino rats weighing 180–200 g. The animals were divided into 12 groups, each containing six animals, and each test sample was given to two groups of rats.	The control and all test groups were orally fed with galactose at a dose of 10 mg/kg body weight. Boswellic acid, corsolic acid, ellagic acid ursolic acid and quercetin were given at a dose of 10 mg/kg body weight. All the animals were sacrificed on the 15th day by spinal nerve dislocation.	All the tested extracts and their active ingredients possess significant the polyol enzyme aldose reductase inhibitory actions with urosolic acid showing the most potent effect. The study indicates the potential of the studied plants and their major constituents as possible protective agents against long-term diabetic complications.	[122]
A total of 48 male Kunming mice were used (four groups, 12 mice in each group).	The aggregated Aβ_25–35_ was injected into the right lateral ventricle with the following coordinates: −0.5 mm anterior/posterior, +1.0 mm medial/lateral and −2.5 mm dorsal/ventral from Bregma (10 nmol in 3 μL of saline per injection). Sham animals were injected in an identical manner with the same amount of sterile saline. Mice were allocated to one of four groups the day after sterile saline or Aβ_25–35_ injection: sham group, Aβ_25–35_-treated group, pinocembrin 20 mg/kg group, and pinocembrin 40 mg/kg group. Pinocembrin was administered by oral gavage once a day continuously for 8 days. The sham group and Aβ_25–35_-treated group received oral gavage in the same manner using distilled water containing 20% hydroxypropyl-β-cyclodextrin without pinocembrin.	Pinocembrin (a flavonoid abundant in propolis), significantly inhibited the upregulation of RAGE transcripts and protein expression both* in vivo* and* in vitro*, and also markedly depressed the activation of p38 mitogen-activated protein kinase (MAPK)-MAPKAP kinase-2 (MK2)-heat shock protein 27 (HSP27) and stress-activated protein kinase (SAPK)/c-Jun N-terminal kinase (JNK)-c-Jun pathways and the downstream nuclear factor κB (NFκB) inflammatory response subsequent to Aβ-RAGE interaction. Pinocembrin significantly alleviated mitochondrial dysfunction through improving mitochondrial membrane potential and inhibiting mitochondrial oxidative stress, and regulated mitochondrion-mediated apoptosis by restoration of B cell lymphoma 2 (Bcl-2) and cytochrome *c* and inactivation of caspase 3 and caspase 9.	[123]
Zebrafish maintenance and experimental procedures were approved by the Committee of Animal Care and Use of Yeungnam University (Gyeongsan, South Korea). Each group (*n* = 70) consumed the designated diet (20 mg/day/fish).	A high cholesterol (HC) diet containing 4% cholesterol was made by soaking tetrabit [Tetrabit Gmbh D49304; 47.5% crude protein, 6.5% crude fat, 2.0% crude fiber, 10.5% crude ash, containing vitamin A (29,770 IU/kg), vitamin D3 (1860 IU/kg), vitamin E (200 mg/kg), and vitamin C (137 mg/kg); (Melle, Germany)] in a solution of cholesterol in diethyl ether. After ether evaporation, HC diet was mixed with lyophilized fruit extract (a final concentration of 10% w/w of powder/ tetrabit). The animals were divided into 5 groups: normal diet (ND) group, high cholesterol (HC) diet group, HC + LL (loquat leaves) group, acai-fed group (HC + acai) and HC + GS (grape skin) group.	Serum glucose levels increased in the high cholesterol diet group, to threefold above the ND group; GS and LL feeding elicited the greatest reduction in hyperglycemia. The groups consuming acai and LL showed much less hepatic inflammation, as well as attenuation of fatty liver and a reduced content of oxidized species.	[124]
A total of 15 male inbred C57BL/6 J mice were used (3 groups, 5 mice in each group).	Diabetes was induced in the mice by a single dose of STZ (200 mg/kg). Mice were fed a normal rodent chow diet (Clea Japan) for 1 week after induction of diabetes. At this time, these mice were administered epicatechin (500 mg/kg/day) orally every day for 45 days. Animals were divided into groups: control group, mice treated with epicatechin (500 mg/kg/day), and mice treated with arbutin, a catechol analogue (500 mg/kg/day).	Administration of 500 mg/kg/day epicatechin to STZ-induced diabetic mice enhanced the CML accumulation on the surface of gastric epithelial cells, whereas administration of 500 mg/kg/day arbutin to STZ-induced diabetic mice did not enhance CML accumulation compared to untreated mice. High amounts of catechol-containing structures enhance oxidative stress, thus leading to enhanced CML formation, and this phenomenon may explain the paradoxical effect that some flavonoids have on redox status.	[111]
Adult male Wistar rats of body weight 150–160 g were used in the study. The animals were divided into four groups of six rats each.	Control animals (CON) received the control diet containing starch and tap water *ad libitum.* Fructose-fed animals (FRU) received the high fructose diet and water *ad libitum.* Fructose-fed animals (FRU-CA) received the high fructose diet and water *ad libitum* and were administered 300 mg carnitine (CA)/kg b.w/day; i.p. Control animals (CON-CA) received the control diet and water *ad libitum* and were administered with 300 mg CA/kg b.w/day; i.p.	The levels of glucose, fructose and fructosamine in plasma and glycated haemoglobin and methyl glyoxal in blood were significantly higher in fructose-fed animals than in the control rats. Administration of CA along with the fructose diet reduced these levels significantly. In rats fed control diet, administration of CA did not produce significant alterations in the parameters when compared with the control group. The rats fed fructose diet showed increased total collagen and glycation in tail tendon and skin as compared to control rats. CA-administered fructose-fed rats registered near-normal levels of collagen and glycation. No significant changes were observed in control rats treated with CA.	[84]

## 6. Conclusions

Many natural compounds, especially polyphenols, have been found to inhibit efficiently protein glycation* in vitro*. Their action* in vivo* is more problematic due to the bioavailability problems. Nevertheless, some positive effects of natural antioxidants against the consequences of excessive glycation have been found. While the mechanisms of their action may go beyond direct inhibition of glycation, there are reasons to expect that natural compounds used as food additives may prevent adverse effects of protein glycation and, in consequence, delay ageing. Another useful approach may consist in limitation of AGE intake in the food.
